# The Relationship Between the Retromandibular Vein and the Extratemporal Segment of the Facial Nerve: A Prospective Cadaveric Study of 24 Hemifaces

**DOI:** 10.7759/cureus.59637

**Published:** 2024-05-04

**Authors:** Alexandros Poutoglidis, Stefanos Triaridis, George K Paraskevas, Paraskevi Karamitsou, Ioannis Mykoniatis, Georgios Langas, Stavros Tsiakaras, Nektarios Galanis, Nikolaos Lazaridis

**Affiliations:** 1 Department of Anatomy and Surgical Anatomy, School of Medicine, Aristotle University of Thessaloniki, Thessaloniki, GRC; 2 First Department of Otorhinolaryngology-Head and Neck Surgery, School of Medicine, Aristotle University of Thessaloniki, AHEPA University Hospital, Thessaloniki, GRC; 3 Department of Otorhinolaryngology-Head and Neck Surgery, 'G. Papanikolaou' General Hospital, Thessaloniki, GRC; 4 First Department of Urology, School of Medicine, Aristotle University of Thessaloniki, ‘G. Gennimatas’ General Hospital, Thessaloniki, GRC; 5 First Department of Urology, School of Medicine, Aristotle University of Thessaloniki, 'G. Gennimatas' General Hospital, Thessaloniki, GRC

**Keywords:** parotid gland, facial nerve, cadaver, balkan peninsula, anatomists

## Abstract

Introduction: Anatomical preservation and functional integrity of the facial nerve (FN) are the main concerns of parotid surgery. Even though a variety of anatomical landmarks have been proposed and widely utilized, temporal or permanent postoperative FN palsy is still a significant comorbidity of parotid surgery. Therefore, the literature must fully elucidate the consistency of the anatomical relationship between the FN and the retromandibular vein (RMV).

Methods: We conducted a cadaveric study of 24 hemifaces to map the relationship between the FN and the RMV. Three distinct patterns were identified. Fourteen of the hemifaces were males, and 10 were females. Thirteen cadaveric dissections were performed on the right side and 11 on the left side.

Results: Our study found three distinct patterns and proposed a classification system. Type I (66.7%) is when the nerve lies exclusively lateral to the RMV. Type II (29.2%) is when the FN lies superficial to the RMV, but its mandibular branch lies deep to the anterior branch of the RMV, and type III (4.1%) is when the FN lies exclusively medial to the RMV.

Conclusion: The FN and RMV relationship is not constant, and surgeons should be aware of every anatomical variation. Especially in cases where the FN is estimated to lie more in-depth to the level of the RMV, a retrograde approach may be required to avoid a FN injury.

## Introduction

Anatomical preservation and functional integrity of the extracranial segment of the facial nerve (FN) are of paramount importance concerning parotid surgery. Any inadvertent injury may cause devastating complications with functional and aesthetic impacts on the patient’s quality of life. The mapping of the FN and the development of surgical anatomical landmarks offer intraoperative guidance to the surgeon and facilitate nerve preservation [1].

In 1956, Davis et al. proposed a classification system of six distinct distribution patterns for the extratemporal segment of the FN [2]. This classification demonstrated the wide anatomical complexity of the FN branching with multiple anastomoses among its final branches. A systematic review validated the results of Davis et al. [3]. Hegazy distinguishes three temporal branches of the FN that pass on the superficial side of the zygomatic arch and deep to the subcutaneous tissue and the temporal fascia [4]. 

The surgical landmarks for the anterograde dissection are well-known (tragal pointer, tympanomastoid suture, posterior belly of the digastric muscle, and styloid process). The anatomical position of the retromandibular vein (RMV) regarding the nerve may render the effort to dissect the FN challenging [5,6]. In most cases, the FN and its two main branches (temporofacial and cervicofacial) lie superficial to the RMV.

The objective of our study is to evaluate the relationship of the extracranial FN and its main branches with the RMV and its division (anterior and posterior branches) in cadaveric specimens.

## Materials and methods

We performed a prospective study at the Laboratory of Anatomy and Surgical Anatomy of the Aristotle University of Thessaloniki in Greece which included 24 consecutive hemifaces of embalmed cadavers. We excluded three cadavers due to irreversible FN damage. As part of a dissertation, the study was prospectively approved by the Institutional Review Board of the Aristotle University of Thessaloniki (No 3607, dated 23/12/2021). Cadavers were dissected meticulously in a retrograde fashion after detecting the marginal mandibular nerve or a buccal branch. Observations and measurements were conducted by two well-experienced anatomists. The specimens were from the Balkan population and donated to the School of Medicine of the Aristotle University of Thessaloniki for educational purposes.

After meticulous dissection of the extratemporal segment of the FN under loop magnification, a visual examination was performed to record the relationship between the FN and the RMV. The course of the FN and the RMV with their respective branches was classified into distinct patterns. No pathology in the examined hemifaces was encountered to disturb the natural relationship between the FN and the RMV. Analysis and comparisons between sexes and the presence of bilateral symmetry were also recorded. Eventually, we documented photographically the most characteristic cases and collected the data in an Excel (Microsoft Corporation, Redmond, USA) file to perform further statistical analysis. 

## Results

**Figure 1:**
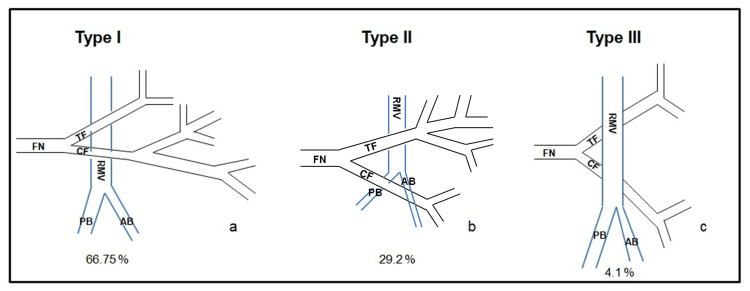
Classification system A classification system (three patterns) is proposed according to the correlation of the course of the FN to the course of RMV and its main branches. RMV: retromandibular vein, AB: anterior branch of the retromandibular vein, PB: posterior branch of the retromandibular vein, FN: facial nerve, TF: temporofacial branch of the facial nerve, CF: cervicofacial branch of the facial nerve

Our study included 24 hemifaces of cadaveric specimens. Fourteen of them were males, and 10 were females. Thirteen cadaveric dissections were performed on the right side and 11 on the left side. Three distinct patterns of the relationship between the FN and RMV were identified. We proposed a classification system that correlates the course of the FN to the course of RMV and its main branches. The three classification patterns can be seen in Figures 1a-1c. 

**Figure 2:**
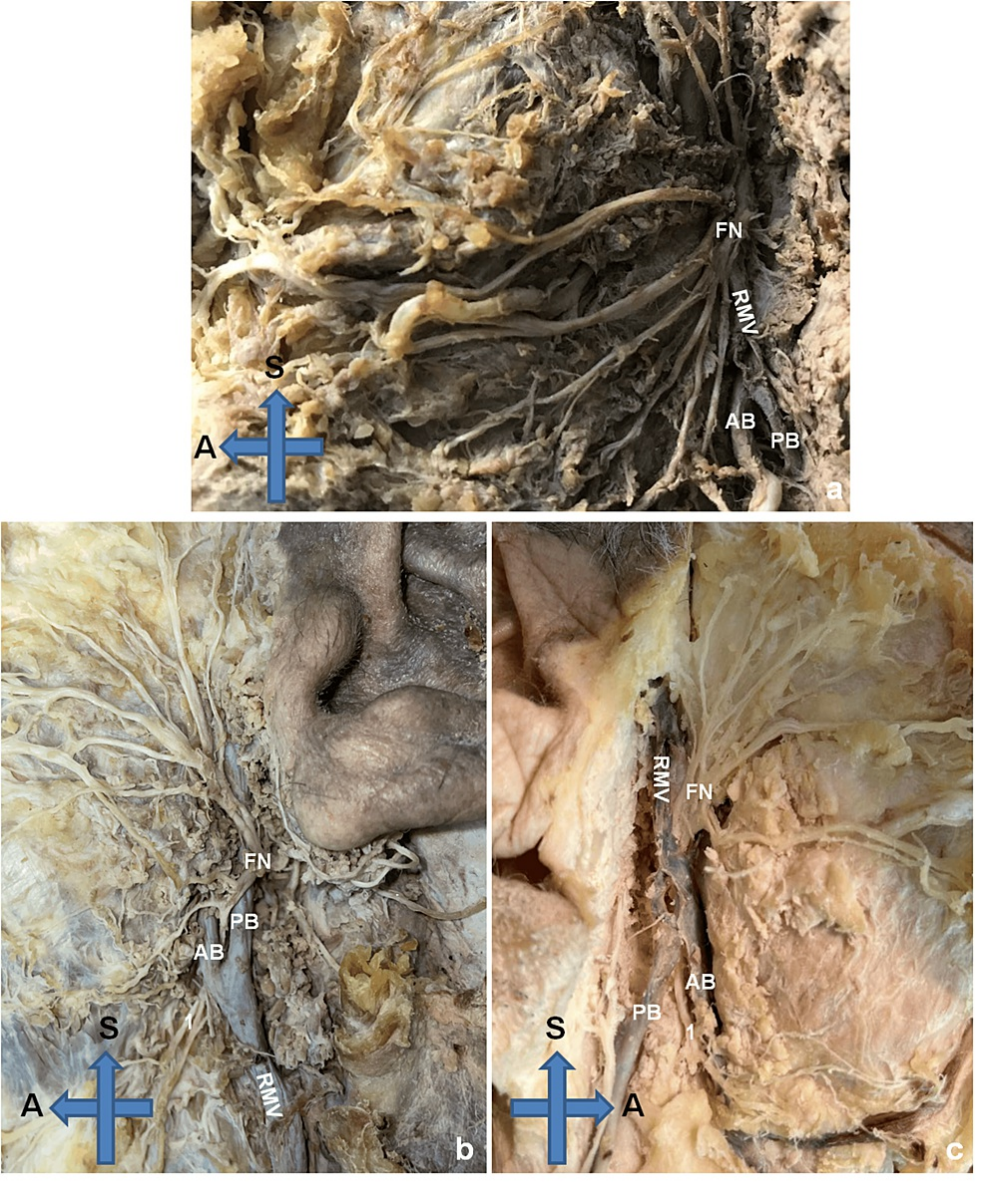
Anatomic specimens Three anatomic specimens are provided. a. Type I: the FN lies exclusively lateral to the RMV, b. Type II: the FN lies lateral to the RMV and its mandibular branch lies deep to the anterior branch of the RMV, c. Type III: the FN lies exclusively medial to the RMV. RMV: retromandibular vein, AB: anterior branch of the retromandibular vein, PB: posterior branch of the retromandibular vein, FN: facial nerve, 1: mandibular branch of the facial nerve, S: superior, A: anterior

The most dominant pattern was type I, where the FN lies lateral to the RMV and its main branches in 16 cases (66.7%). The second most common pattern was type II, where the FN lies lateral to the RMV, but its mandibular branch lies deep to the anterior branch of the RMV with 29.2% (seven cases). Finally, we encountered one case (4.1%) with a type III pattern, where the FN was exclusively deep to the RMV (Figure 2). 

Male specimens were classified as a type I pattern in nine cases, type II in four cases, and type III in one case, while female specimens were classified as type I in seven cases and type II in three cases. The distribution among the sexes did not demonstrate any statistically significant differences (p>0.005). 

As for the right side, type I was recorded in nine hemifaces, followed by type II with three hemifaces and type III with just one case. On the left side, type I was recorded in seven hemifaces, and type II was recorded in four hemifaces. There were also no statistically significant differences among sides.

As for the bilateral symmetry, we examined 10 cadavers. On half of the cadavers, similar patterns were detected on both sides.

## Discussion

Parotid surgery is considered a challenging procedure due to the proximity of parotid tumors to the FN [7]. Surgeons tend to use nerve monitoring for the safe identification and dissection of the FN [8]. As in every surgery, anatomical landmarks have been utilized to assist surgeons’ efforts to preserve the integrity of the FN.

In 2018, Ji et al. presented a systematic review of studies measuring the distance of the FN to the prominent anatomical landmarks [1]. According to their results, the tragal pointer was 12.5-14.7 mm away from the FN, the posterior belly of the digastric was 4.80-12.78 mm away from the FN, and the tympanomastoid suture was 9.80 mm lateral to the FN. These results demonstrated the inconsistent position of the FN with the tragal pointer [9,10].

In our study, the FN was lateral to the RMV in most cases. Our results agree with the available literature [11]. The RMV is the radiologic anatomical landmark dividing the parotid gland into superficial and deep lobes. Many authors also consider the RMV as the radiologic landmark to localize the FN in imaging [12-14]. However, no radiologic study can directly identify the FN and determine its position compared to the RMV.

The literature needs more quality anatomical studies describing the variations of the relation between the RMV and the FN [15,16]. Surgical studies are mostly about cases with parotid tumors. High-volume neoplasms tend to dislocate the FN and RMV, modifying their normal relationship. In their studies, Laing et al. and Kopuz et al. agreed that the FN was lateral to the RMV in 90% of cases [15,16]. Toure and Vacher reported the FN to lie medial to the RMV in 13.6% of cases [11]. The reported studies of these variations are extremely rare [17,18].

In 2022, Khan et al. presented four characteristic variations in the RMV that caused difficulty during FN dissection [19]. In the first case, the FN was lying between the two main branches of the RMV division, and it could be associated with type II of our classification. In the fourth case, the nerve lies more profound than the RMV, rendering anterograde dissection extremely difficult. This case could be classified as type III according to our classification system.

The current study is in the light of certain limitations. First of all, it is a cadaveric study and the post-mortem tissue changes may contribute to a relevant dislodgement of the FN and RMV. Secondly, the vascular properties tend to have certain changes in embalmed cadavers. However, the study included adequate specimens to propose a classification system; there are always extremely rare variations and anomalies that cannot be classified in any classification system. The results should be validated with a study of a larger series.

## Conclusions

The relationship between the FN and the RMV was always debatable. As proven in the current study, the nerve mostly lies lateral to the RMV making anterograde dissection feasible. However, in cases where the nerve lies medial to the RMV or runs between the divisions of the RMV, a retrograde dissection may be required to avoid potential FN injury. We proposed a classification pattern to assist surgeons during parotid surgery by providing valuable knowledge of the relationship patterns between the FN and RMV. Surgeons should be competent in performing both anterograde and retrograde dissections to facilitate intraoperative transition if required during surgical management of challenging parotid lesions. 
